# *In vitro* assays for investigating the FLASH effect

**DOI:** 10.1017/erm.2022.5

**Published:** 2022-02-28

**Authors:** Gabriel Adrian, Jia-Ling Ruan, Salomé Paillas, Christian R. Cooper, Kristoffer Petersson

**Affiliations:** 1 Division of Oncology and Pathology, Clinical Sciences, Skåne University Hospital, Lund University, Lund, Sweden; 2 Radiation Physics, Department of Haematology, Oncology and Radiation Physics, Skåne University Hospital, Lund University, Lund, Sweden; 3 MRC Oxford Institute for Radiation Oncology, University of Oxford, Old Road Campus Research Building, Oxford OX3 7DQ, UK; 4 Leicester Cancer Research Centre, University of Leicester, Robert Kilpatrick Clinical Sciences Building, Leicester Royal Infirmary, Leicester LE2 7LX, UK

**Keywords:** Cell models, cells, dose rate, radiation, radiotherapy, ultra-high

## Abstract

FLASH radiotherapy is a novel technique that has been shown in numerous preclinical *in vivo* studies to have the potential to be the next important improvement in cancer treatment. However, the biological mechanisms responsible for the selective FLASH sparing effect of normal tissues are not yet known. An optimal translation of FLASH radiotherapy into the clinic would require a good understanding of the specific beam parameters that induces a FLASH effect, environmental conditions affecting the response, and the radiobiological mechanisms involved. Even though the FLASH effect has generally been considered as an *in vivo* effect, studies finding these answers would be difficult and ethically challenging to carry out solely in animals. Hence, suitable *in vitro* studies aimed towards finding these answers are needed. In this review, we describe and summarise several *in vitro* assays that have been used or could be used to finally elucidate the mechanisms behind the FLASH effect.

## Introduction

Recent preclinical studies have shown that FLASH irradiation, which is radiation delivered in a fraction of a second, reduces incidence and severity of radiation side effects compared to conventional dose rate (CONV) irradiation used in clinical practice (Refs 1–16). However, the treatment effect on tumours is not reduced (Refs 1, 5, 11, 12, 17, 18). This has been called the ‘FLASH effect’. The FLASH sparing effect has mainly been observed using relatively large single doses *in vivo*, though a few studies have shown an effect also *in vitro* (Refs 7, 8, 19–22). The benefit of FLASH radiotherapy (RT) has been further shown in veterinary clinical studies and in the first treatment of a human (Refs 6, 10, 23). FLASH-RT is delivered with irradiation systems with a high radiation output, capable of generating the ultra-high dose rates and short delivery times required for producing an observable FLASH sparing effect, which permit treatments to be delivered in fractions of a second, compared to several minutes for conventional treatments (Refs 4, 24–26).

**Fig. 1.**
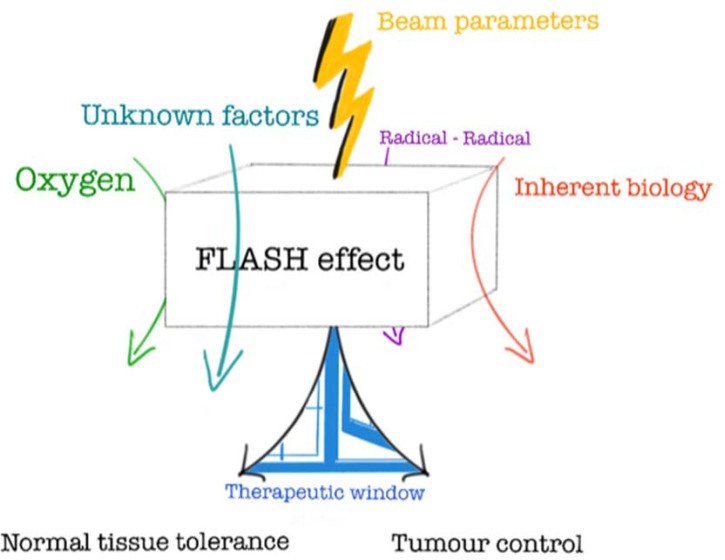
‘The FLASH box’ - Illustrating what we know and what is yet to be discovered about the radiobiological mechanisms behind the highly beneficial ‘FLASH effect’ © Gabriel Adrian.

The short treatment times used in FLASH-RT, often less than 0.1 s, have the added value of minimising treatment delivery uncertainties caused by patient motion during delivery, for example, reduced risk of missing a lung tumour due to the breathing motion. The potential to ‘freeze’ physiological motion could allow for the use of smaller motion-related target margins, and thereby smaller volumes of normal tissue being unnecessarily irradiated. Due to these advantages, FLASH-RT has the potential to be an important (r)evolutionary step in cancer treatment (Refs 16, 27). However, the radiobiological mechanisms responsible for this differential FLASH sparing effect observed between normal tissue and tumour tissue are not yet known, though several hypotheses have been proposed (Fig. 1) (Ref. 16), e.g. radiochemical depletion of oxygen leading to transient hypoxia (Refs 28–31), radical-radical interaction (Refs 32, 33), and a modified immune response following FLASH relative to CONV irradiation (Refs 34, 35).

For an optimal translation of FLASH-RT into clinical trials, it is essential to know what specific beam parameters induces a FLASH effect and what the radiobiological mechanisms involved are. The FLASH effect is currently considered by many as an effect only seen *in vivo*. However, elucidating the mechanisms underpinning the FLASH sparing effect solely utilising *in vivo* models may prove to be difficult and ethically unviable. Hence, suitable *in vitro* studies are needed, aimed towards finding optimal beam parameters and pin-pointing the biological mechanisms. Here, we describe several *in vitro* assays that have been used in preclinical FLASH studies as well as other assays that could be used to fully understand and exploit the benefits associated with FLASH-RT.

## Clonogenic assays

In radiobiological research, the clonogenic assay has been a reference method for *in vitro* studies since Puck & Markus introduced it in 1956 (Ref. 36). Radiation-induced cell death can occur in several ways, including mitotic catastrophe, apoptosis, necrosis, senescence, autophagy and ferroptosis (Ref. 37). The advantage of the clonogenic assay is its ability to capture cancer cells ability (or inability) of ‘endless’ division, i.e. including all kinds of cell deaths. Hence, the assay provides an *in vitro*-surrogate for the complete sterilisation of tumour cells *in vivo*. Although the assay may have limitations, such as being dependent on cell densities, it should be a reliable method for head-to-head comparisons in dose rate studies, provided all other experimental conditions are equal (Refs 38–40).

Interestingly, both early (from the 60's) and recent (here defined as in the 21st century) studies of ultra-high dose rate irradiations in mammalian cells using clonogenic assays have provided inconsistent results. In the early days of ultra-high dose rate irradiation, a ‘hockey-stick’-shaped survival curve for ultra-high dose rate irradiated samples was described. Typically, CONV and FLASH-curves were indistinguishable at lower doses, and then separated at higher doses, i.e. an increased survival fraction after FLASH irradiation was noted at higher irradiation doses (Refs 41, 42). Such survival curves were initially described for normoxic conditions (ambient air, 21%), though other experiments could not reproduce the findings (Refs 43, 44). Attention was then turned to the dependence of oxygen. It was found that the ‘break’ of the survival curve was influenced by the oxygen tension (Refs 43–46). Increasing the level of hypoxia in cells resulted in a lower total dose required to ‘break’ the survival curve. In addition to these results, similarly shaped survival curves were also described when irradiating bacteria at ultra-high dose rates, with breaks at doses around 60–70 Gy in normoxic conditions (Refs 47, 48). During the 90's, two independent studies could not detect any survival difference after FLASH and CONV irradiation in neither normoxic nor anoxic conditions (Refs 49, 50).

Recent investigations using clonogenic assays in normoxic conditions have also provided inconsistent results. Despite the fact that the FLASH sparing *in vivo* is found in the healthy tissue, few of the recent studies have used normal cell lines. Instead, most have investigated the potential sparing of cancer cell lines. An increased survival fraction was found for H454 murine glioblastoma cells after 20 Gy FLASH compared with CONV irradiation in normoxia (Ref. 8). Congruently, an increased survival fraction was found for 4/7 cell lines after FLASH compared with CONV in our recent investigations (Ref. 51). On the other hand, Venkatesulu *et al*. found opposing FLASH effects, hence a lower survival fraction after FLASH for two murine pancreas cancer cell lines in normoxia (Ref. 52). Other studies could not distinguish any differences in survival fraction in normoxia for IMR90 normal human lung fibroblasts, DU145 prostate cancer cells, or A549 lung cancer cells (Refs 19, 20, 22). In hypoxia, we have previously described an oxygen-dependent FLASH sparing for DU145 prostate cancer cells (Ref. 19). Similarly, A549 lung cancer cells irradiated as spheroids with naturally occurring hypoxic cores exhibited a FLASH sparing, in contrast to the results for cells irradiated as a normoxic monolayer (Ref. 22). Noteworthy, in the early publications, the dose required to break the survival curve under normoxia was found to be at 7–10 Gy with a distinct inflexion point (Refs 41, 42). Such inflexion points have since only been described for cells in hypoxia and/or for much higher (non-clinical) doses and seem to indicate the dose required to consume all available oxygen (Ref. 46). Later studies have instead successfully used the linear-quadratic (LQ) model to fit the data (Ref. 53).

The reasons for the inconsistent findings of FLASH *versus* CONV using clonogenic assays could be several (Table 1). Firstly, we would not expect to see significant oxygen depletion effects for FLASH *versus* CONV in fully anoxic cell cultures as there is no oxygen available to deplete, nor in normoxic cultures as there is too much oxygen to deplete for clinical doses to have a meaningful impact on the available oxygen. Furthermore, beam characteristics differ between laboratories. Single-pulse *versus* pulsed delivery, instantaneous dose rate, dose per pulse, pulse repetition frequency, average dose rate, total delivery time and type of irradiation (electron/photon *versus* ion) could possibly all affect the radiobiological response (Refs 5, 16). Experimental conditions vary between laboratories. Temperature during irradiation, time outside the incubator, type of cell medium used, the volume of cell medium per flask or dish might influence results (Ref. 54). The definition of survival is stated to be a single cell that has proliferated to form a colony of at least 50 cells (Ref. 36). Laboratories may use different approaches to determine the colony size, i.e. to decide which clones are to be counted as survivors. It has been shown that different clone-size cut-offs influences the results in clonogenic assays (Refs 55–57).

**Table 1.** Published Clonogenic FLASH data using mammalian cells, separated by electron and other type of irradiation

Publication	Year	Cell line	Plating method	Dose-range (Gy)	Average dose rate for a 10 Gy delivery (Gy/s)	Instantaneous dose rate (Gy/s)	Dose/pulse (Gy)	Pulse repetition frequency (Hz)	Total delivery time	Energy	Type of irradiation	FLASH sparing normoxia	FLASH sparing hypoxia
Town (Ref. 42)	1967	HeLa S3	post	0–25	7 × 10^6^ or 4 × 10^3^	<3.5 × 10^7^	single or two pulse(s)	single pulse or 400	1.3 *μ*s or 2.5 ms	15 MeV	electron	yes	N/A
Nias *et al*. (Ref. 58)	1969	HeLa	post	0–27	1 × 10^7^	1 × 10^7^	single pulse	single pulse	1 *μ*s	8–14 MeV	electron	N/A	probably, but no direct comparison
Nias *et al*. (Ref. 44)	1970	HeLa	post	0–33	1 × 10^9^	~10^9^	single pulse	single pulse	10 ns	10 MeV	electron	N/A	N/A
Epp *et al*. (Ref. 45)	1972	HeLa-S3	pre	0–35	3 × 10^9^	~10^9^	single pulse	single pulse	3 ns	350 keV	electron	N/A	probably, but no direct comparison
Michaels *et al*. (Ref. 46)	1978	CHO	pre	0–45	3 × 10^9^	~10^9^	single pulse	single pulse	3 ns	600 keV	electron	no	probably, but no direct comparison
Zackrisson *et al*. (Ref. 50)	1991	V79–379-A	post	0–40	380	2.7 × 10^5^	1.6	200	~30 ms (10 Gy)	50 MeV	electron	no	no (anoxic)
Cygler *et al*. (Ref. 49)	1994	U87-MG, HT-144	post	0–27	3 x10^6^	~ 5 × 10^6^	single pulse	single pulse	3.2 *μ*s	20 MeV	electron	no	no (anoxic)
Montay-Gruel *et al*. (Ref. 8)	2019	H454	post	20	1000	1.8 × 10^6^	6.7	100	20 ms	6 MeV	electron	yes	yes
Venkatesulu *et al*. (Ref. 52)	2019	KPC, Panc02	pre	0–8	35	–	–	–	–	20 MeV	electron	reversed	N/A
Adrian *et al*. (Ref. 19)	2019	DU145	pre	0–25	800	8.6 × 10^5^	3	200	~10 ms (10 Gy)	10 MeV	electron	no	yes
Adrian *et al*. (Ref. 51)	2021	MCF7, MRC-5, Lu-HNSCC4, MDA-MB-231, HeLa, WiDr	pre	0–12	800	8.6 × 10^5^	3	200	~10 ms (10 Gy)	10 MeV	electron	yes	N/A
Khan *et al*. (Ref. 22)	2021	A549, MDA-MB-231, HT29	post	0–20	90	4.0 × 10^5^	1	90	~100 ms (10 Gy)	16 MeV	electron	no	yes
Berry *et al*. (Ref. 41)	1969	HeLa S3	post	0–15	~ 1 × 10^9^	~10^9^	single pulse	single pulse	7 ns	2.0 MVp	photon	yes	N/A
Auer *et al*. (Ref. 59)	2011	HeLa-RIKEN	post	3	–	10^9^	3	–	< 1 ns	20–25 MeV	proton	no	no
Pommarel *et al*. (Ref. 60)	2017	HCT116-WT, HCT116-p53-/-	post	0–10	–	9 × 10^7^	1.15	N/A	–	5 MeV	laser-accelerated protons	no	no
Manti *et al*. (Ref. 61)	2017	HUVEC	–	0–5	–	–	N/A	N/A	–	–	laser-accelerated protons	no	N/A
Bayart *et al*. (Ref. 62)	2019	SF763, U87	post	0–10	–	1.5 × 10^8^	0.72	N/A	–	–	laser-accelerated protons	no	N/A
Buonanno *et al*. (Ref. 20)	2019	IMR90	post	0–10	1000	–	–	–	–	5.5 MV	protons	no	N/A
Tessonnier *et al*. (Ref. 63)	2021	A549, H1437	post	0–12	~185(±35)	–	N/A	N/A	14–86 ms	145.74 MeV/u	Helium ions	no	yes

**Fig. 2.**
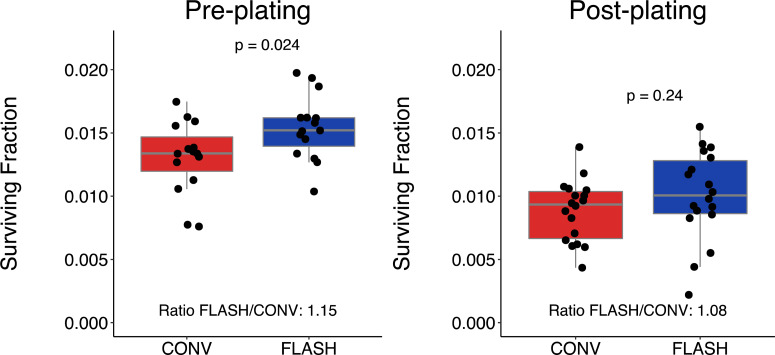
Clonogenic survival for the melanoma cell line MM576 after irradiation with 9 Gy of CONV (red, measured delivered doses 9.2–9.4 Gy) or FLASH (blue, measured delivered doses 9.4–9.6 Gy) using the pre-plating (left panel) or post-plating method (right panel). The box and whisker plots illustrate median (grey line), interquartile range (box), the lowest/highest observation within ± 1.5 × interquartile range from the box (whiskers), and individual flasks as black dots. Irradiation was performed using a modified clinical accelerator (Ref. 28) with beam characteristics and the experimental pre-plating protocol as previously described (Ref. 14). For the post-plating, 500 000 cells were plated in T12.5 flasks the day before irradiation, and 1 h after irradiation the cells were trypsinised, counted, re-plated in appropriate densities and then incubated and evaluated as the pre-plating flasks. Statistical comparisons were made in RStudio (version 1.2.5042) using the unpaired Student's *T*-test after testing for normality using the Shapiro-Wilk's test. ‘Ratio FLASH/CONV’ was calculated as the mean (Survival Fraction_FLASH_) divided by the mean (Survival Fraction_CONV_). Data from three independent experiments with triplicate-sextuplicate flasks per condition.

Challenges also arise from performing the clonogenic assay with irradiation at higher total doses. Depending on the cell line's plating efficiency and radiation sensitivity it may not be possible to achieve more than a few surviving colonies. Thereby, statistical uncertainties arise. In addition, the high inoculation density may cause difficulties to evaluate the samples. Together, clonogenic assays performed at higher dose ranges may be less reliable. The number of plated cells per flask or dish, the cell density, also affects the radiobiological response (Refs 38–40). In addition, the clonogenic assay can be performed in two principally different ways; pre-plating or post-plating (Ref. 64). In the pre-plating method, cells are plated in appropriate densities in individual flasks, dishes or wells, and each sample is irradiated. The cells are then incubated, without re-trypsinisation, to grow and form colonies. In the post-plating methods, a densely seeded flask is irradiated, trypsinised, cells are counted, plated in appropriate cell densities in dishes and allowed to grow and form colonies. Depending on the method used, different radiation response has been reported (Ref. 65). To investigate if plating methods could influence some of the reported inconsistent FLASH findings, we have performed pre- and post-plating experiments for the melanoma cell line MM576. Our current results suggest that both plating methods may detect a FLASH sparing, although the magnitude of the sparing and the power to detect statistical differences could differ (Fig. 2). Our lab has investigated clonogenic survival in normoxia after FLASH and CONV for nine human cell lines (including the MM576 in this publication) (Refs 19, 51). Five of the nine cell lines were found to have significantly increased survival fractions after FLASH irradiation. Although it is possible that statistical uncertainty caused the observed discrepancy between cell lines, it is also possible that a difference in biological factors affected the FLASH response. It has been found that the response to FLASH irradiation *in vivo* is cell line specific (Ref. 66), which is likely to also be the case *in vitro*. Further studies comparing different experimental set-ups could reveal additional phenomenological insights of the radiation response. For instance, by varying the time before re-plating after irradiation (delayed plating), the effect of ‘potentially lethal damage’ could be assessed (Ref 67).

Despite potential pitfalls with the clonogenic assay, it is still considered as the ‘gold standard’ and will undoubtedly help verify important findings in future FLASH studies. If care is taken to ascertain identical experimental conditions (e.g. time outside the incubator, cell medium volume, cell density and well-matched CONV and FLASH beam characteristics and irradiation doses), the assay will provide robust and reproducible data. For instance, the importance of beam line characteristics for the FLASH effect, such as pulse repetition frequency, dose per pulse, total delivery time, and average dose rate can be investigated in large-scale experiments. Detailed studies of the FLASH-response in physoxic and/or hypoxic/anoxic conditions (physoxia: oxygen levels of 3–7%, hypoxia: oxygen level ⩽2%, anoxia: 0% oxygen (Ref. 68)) are possible using the clonogenic assay, if care is taken to control the oxygen tension at the time of irradiation. Confirmatory studies in labs with different beam lines, using the same cell line, will be important. Besides varying oxygen concentration, the addition of scavengers such as superoxide dismutase might provide indirect mechanistic evidence for the underlying mechanism of the FLASH effect.

The current data suggest that the clonogenic assay can resolve at least part of the FLASH effect. Due to the clonogenic assay's central role in radiobiological research and the great advantage of large-scale experiments without involving living mammals, the continued use of clonogenic assays to investigate the FLASH effect will be important in our search for the underlying mechanisms.

## Antibody-based assays

Radiation changes the physical and chemical environment of the cell, which leads to changes in protein phosphorylation, enzymatic activity, localisation, and the formation of protein-protein complexes that participate in cell cycle arrest, apoptosis, and DNA repair (Refs 69–72). Ionising radiation can induce DNA damage by direct interaction with the DNA or indirectly through the production of free radicals, which can react with the DNA. This causes a variety of DNA lesion types including base damage, single-strand breaks (SSBs) and double-strand breaks (DSBs) (Refs 73, 74). The generation of these DNA lesions, particularly DSBs, triggers sophisticated and highly regulated DNA damage response and repair (DDR) pathways (Ref. 75) that can easily be assessed *in vitro* by antibody-based techniques such as western blotting (WB), enzyme-linked immunosorbent assay (ELISA), flow cytometry, immunocytochemistry (ICC), or immunohistochemistry (IHC).

Both IHC and ICC provide spatial information such as distribution or localisation of specific cellular or subcellular components. IHC is generally applied on biological tissues from *in vivo* samples either embedded by paraffin or frozen to maintain the morphology, while ICC is generally applied on cells. They are especially useful in studying the DNA damage foci, which are subnuclear foci formed by DDR proteins near the DNA lesion site. IHC has provided some of the recent evidence identifying an *in vivo* FLASH effect. IHC staining for immune, apoptotic, and DDR markers showed compared to CONV irradiation, FLASH irradiation causes less apoptosis, DNA damage, and immune response in guts (Ref. 12), lung (Refs 1, 7), and brain (Refs 8, 9, 15). WB refers to the transfer of biological samples (mixture of proteins from cells or tissue samples) from a gel to a membrane and their subsequent detection on the surface of the membrane. It allows for the semi-quantification of a protein of interest amid a complex protein mixture, which can be achieved by ELISA, involving the use of standard curves with known protein concentrations. While WB detects the protein of interest from a mixture of cells, flow cytometry enables the study of intracellular and cell surface proteins at single-cell resolution.

The phosphorylation of the histone H2AX (*γ*H2AX) is one of the most well-characterised DNA DSB markers. It generally occurs within minutes at the DSB sites in the nucleus and shows a maximum number of foci around 30 minutes post-irradiation (Ref. 76). Therefore, *γ*H2AX can be used as a marker for exploring the spatial and temporal dynamics of DNA repair in cells following irradiation (Refs 77–80). In addition, *γ*H2AX has been suggested as a prognostic biomarker to predict the radiotherapy response of patients (Ref. 81). Another classic DDR marker is 53BP1, which becomes hyperphosphorylated and colocalises with *γ*H2AX near the site of DNA DSBs (Ref. 82).

WB, ICC, and IHC have previously been used to assess DNA damage level after FLASH or CONV irradiation. Using IHC, acute apoptosis was quantified with caspase-3 cleavage and TUNEL labelling in histological sections of irradiated lungs (Ref. 1), brain (Ref. 83) and intestines (Ref. 12). Using flow cytometry, Ehlert *et al*. showed increased *γ*H2AX signal intensity in Jurkat and Ramos cells with increased radiation dose, using laser-accelerated protons at a dose rate of 10^8^ Gy/s (Ref. 84). Fouillade *et al*. (Ref. 7) investigated *γ*H2AX and recruitment of 53BP1 at sites of DNA damage by immunofluorescence microscopy in two normal lung fibroblast cell lines (MRC5 and IMR90) and A549 lung cancer cells. Though no difference in *γ*H2AX foci per nucleus was found among the cell lines between CONV and FLASH irradiation, less 53BP1 was observed in the two normal lung fibroblasts after FLASH irradiation but not in the cancer cell line. However, Adrian *et al*. (Ref. 51) found no significant difference in 53BP1-foci number after FLASH and CONV irradiation for three studied cell lines.

## Comet assay

Another method that has been implemented in many radiobiology studies to measure DNA damage and repair is the comet assay (Refs 85, 86). It was first introduced by Ostling and Johanson using neutral lysis (Ref. 87) in 1984 and then modified by Singh *et al*. to an alkaline version to increase the sensitivity (Ref. 88). The alkaline comet assay is an inexpensive, time efficient, highly sensitive method for assessing DNA damage formation and repair at the level of single cells (Refs 89, 90). In its simplest form, it enables the detection of SSBs, DSBs, alkali-labile sites (ALS), as well as SSB sites associated with incomplete DNA excision repair (Ref. 91). The comet assay can be further extended to detect DNA-DNA/DNA-protein cross-links, adding to the versatility in its applications (Refs 92–96).

In the alkaline comet assay, cells embedded in low melting point agarose on microscope slides are lysed in a solution containing high salt and detergents to remove cell membranes, cytoplasm, nucleoplasm and histones leaving nucleoids of supercoiled DNA linked to the nuclear matrix (Ref. 97). Relaxed in alkaline buffer, broken negatively charged DNA is free to migrate towards the anode upon electrophoresis. These stretched nucleoids resemble comets upon staining with an intercalating DNA dye which may be visualised via fluorescence microscopy (Ref. 89). The intensity of the comet tail relative to the amount of DNA residing in the ‘head’ indicates the level of induced strand breaks; with a reduction in DNA migration indicative of DNA crosslinks (Ref. 91). Furthermore, the addition of lesion-specific bacterial repair enzymes post-lysis enables the detection of a variety of DNA lesions other than the standard SSBs, DSBs, and ALSs (Refs 98–100). Therefore, the assay is useful in the profiling of DNA damage following different delivery modalities of ionising radiation, for instance different dose rates or beam particles.

The comet assay may also be used as a diagnostic, prognostic, and predictive biomarker in oncology (Refs 101, 102). It has been used as a tool for predicting an individual's tumour sensitivity to radiation with and without chemotherapeutic regimes (Refs 85, 103–105). A key feature of the assay is that only a small cell sample is required, making it possible to analyse cells from biopsy prior to and post treatment (Refs 101, 104), or from lymphocyte cells obtained via finger prick (Refs 106, 107). Historically, the assay has been used to identify the hypoxic fraction of tumours in mice and humans obtained from fine-needle aspiration (Refs 108, 109). This method also allows for monitoring changes to the hypoxic fraction of cells during a course of fractionated RT (Ref. 108).

The comet assay has recently been used by us to assess the difference in DNA damage formation of human peripheral blood lymphocytes (PBL) irradiated with 6 MeV electrons at FLASH (2 kGy/s) or CONV (0.1 Gy/s) dose rates, under a variety of oxygen concentrations. This was achieved by incubating cells embedded in low melting point agarose gels mounted on glass slides for 2 h in a humidified hypoxia chamber, prior to irradiation. PBL were used as a representative body-wide systemic normal tissue susceptible to irradiation, to assess DNA damage formation following FLASH or CONV irradiations (Refs 110–112). We found that the difference in DNA damage was modulated by the oxygen concentration, with a maximum difference of 30–40% seen at 0.25–0.5% oxygen tension. We also utilised the method to show how dose rate and total dose modulated the *ex vivo* DNA damage sparing effect observed between FLASH and CONV irradiation at a low (0.5%) oxygen tension, with significant sparing observed at average dose rates ⩾ 30 Gy/s and total doses ⩾ 20 Gy (Ref. 113).

## Genomic approach

We and other groups have used RNA sequencing to identify potential novel biomarkers relevant to the differential RT response between FLASH and CONV irradiation (Refs 7, 114, 115). In addition, functional genomic screening using techniques like CRISPR (Ref. 116) or RNA interference (Ref. 117), which provides a large-scale genetic loss-of-function experimental approach, can be used to systematically identify the genes responsible for the FLASH effect. However, such methods are generally very time-consuming, resource demanding, and sometimes challenging to perform as they require high standards in quality control to give robust and reliable results, i.e. it requires identical irradiations (FLASH and CONV) of numerous identically prepared samples. In addition, the results will also need to be carefully validated in subsequent experiments using assays such as the ones described above.

## Assessing oxygen content, free radicals and oxidative stress

It has been observed by us and several other groups that the FLASH effect is oxygen dependent (Refs 8, 19, 29, 63, 118). Therefore, being able to measure and control the oxygen concentration is of importance when conducting FLASH experiments. Techniques to measure oxygen tension in tissues *in vivo* have been previously reviewed (Ref. 119), some of which may be applied *in vitro*. Due to the nature of oxygen depletion during FLASH irradiation, the oxygen measurement technique of choice should ideally have a temporal resolution in the order of milliseconds. Quenching of fluorescent or phosphorescent dyes by oxygen is a common technique that has been applied to measure the oxygen consumption during FLASH irradiation. We and other groups have used fibre optic probes coated with oxygen-sensitive fluorescent compounds to measure the local oxygen tension *in vitro* during FLASH irradiation (Refs 31, 120). Using a water-soluble molecular probe Oxyphor 2P, Cao *et al*. measured and compared the oxygen consumption during FLASH and CONV irradiation, both *in vitro* and *in vivo* (Ref. 121). Other oxygen measurement techniques could be used to study the *in vitro* oxygen dynamic in FLASH irradiation, e.g. cellular tracking of oxygen concentration can be achieved using soluble oxygen probes and fluorescence/phosphorescence lifetime imaging microscopy (Refs 122, 123).

It has been hypothesised that the differential tissue response between FLASH and CONV irradiation is due to difference in damage from radiation-induced free radicals, caused by the higher temporal concentration of radicals produced for FLASH leading to an increase in radical-radical interactions and consequently less indirect damage of DNA (Fig. 1) (Refs 32, 124). Therefore, detection of free radicals in cells and tissues will be important to decipher if this is the mechanism responsible for the FLASH effect. A comprehensive review on free radical detection has previously been published by Halliwell and Whiteman (Ref. 125). Direct measurement of reactive oxygen species (ROS, a subset of free radicals containing oxygen) in cells is generally achieved by fluorogenic probes (Ref. 126). In addition, cells genetically encoded with fluorescent protein-based redox sensors can be used to capture the ROS dynamics within cells, in real-time (Ref. 126). Electron paramagnetic resonance (EPR) is the only technique that can detect specific free radicals directly, and it can also be used to measure oxygen concentration (Ref. 127). However, many of the free radicals generated *in vitro* or *in vivo* have very short half-life times and will require the use of ‘spin-traps’, chemicals that can form stable radicals, to enable detection by EPR. Incorporation of antibody-based technology with EPR, the ‘immuno-spin trapping,’ can detect DNA radicals with high sensitivity and subcellular information (Ref. 128). These techniques allow for spatiotemporal detection of free radicals following *in vitro* FLASH and CONV irradiation.

Compared to detecting free radicals, the oxidative stress, the damage of cells and tissues caused by ROS, is less technically challenging to detect since the markers are generally more stable. Oxidative stress can be assessed by DNA damage, lipid peroxidation, and protein damage using antibody-based or chromatographic assays (Ref. 129). In addition, fluorogenic lipophilic probes have been developed to study the spatiotemporal information of lipid metabolism (Ref. 130).

## Novel *in vitro* assays

The radiobiological assays used for studying the FLASH effect have so far mostly been conducted with 2-dimensional (2D) cell cultures. Recent advancement in 3-dimensional (3D) cultures has offered more ways to model/mimic physiological conditions. Therefore, these technologies may be useful to create second-order events such as oxygen gradient and immune response, which could enhance the FLASH effect. The characteristics of each technology and the potential second-order events each can model to enhance the FLASH effect are summarised in Table 2.

**Table 2.** Novel cell culture technology and the potential secondary events to enhance the FLASH effect

Type	Characteristics	Secondary event		
		Oxygen gradient	Immune effect	Blood flow
Spheroids	Single cell population	Yes	No	No
Organoids	Multi-cell populations Have stem cells Self-renewal (biobanking possible)	Yes	No	No
Tissue slices	Multi-cell populations Recapitulate spatial cellular information	No	Yes	No
Organ-on-chip	Single or multi-cell population Recapitulate mechanical microenvironment High flexibility	Yes	Yes	Yes
Engineered tissues	Single or multi-cell population High flexibility	Yes	Yes	Yes

### Spheroids

Tumour spheroids are aggregates of cancer cells with 3D cell-cell contact. They can be either derived by permanent cancer cell lines or patient-derived tumour cells. Generation of spheroids by mixing cancer cells with other cell types (e.g. stromal cells) is also possible and widely used in drug screening. The 3D geometry of the tumour spheroids allows for the generation of oxygen and nutrient gradients from the outer layers to the centre, similar to *in vivo* tumours though being avascular. The outer cells of the tumour spheroid are generally proliferating, and the inner quiescent or sometimes necrotic. The application of tumour spheroids in radiobiology can be found from an earlier review by Santini *et al*. (Ref. 131). However, the potential of this technology was not fully realised until the recent development of high throughput imaging and high content screening (Ref. 132). Compared to *in vivo* models, tumour spheroids present a simple but still physiologically relevant model for radiobiology as their radiation response is independent of vasculatures, the normal tissues, and the immune cells. Therefore, the model can be used to understand the direct radiation response of cancer cells under simple environmental cues of oxygen and nutrients. With its spherical geometry, the environmental gradient and growth of tumour spheroids can be modelled *in silico* (Refs 133, 134), making it an easy model to be utilised with radiochemical or radiobiological modelling.

Use of tumour spheroids as a model for oxygen depletion during FLASH irradiation has been reported by Khan *et al*. (Ref. 22). Tumour spheroids were irradiated before they formed necrotic cores. After irradiation, tumour spheroids were subsequently dissociated for clonogenic assay. Using this method, they showed that irradiating tumour spheroids can result in a large difference in cell survival between FLASH and CONV irradiation, with a maximum dose-modifying factor of 1.3, while they observed no difference in survival in 2D cell culture. The enhanced FLASH effect in tumour spheroids is likely caused by the relative increase in the hypoxic cell population during the FLASH irradiation, due to radiochemical oxygen depletion, and the differential radiosensitivity of normoxic and hypoxic cells within the spheroids. Interestingly, their study also showed that the growth kinetics of the intact spheroids is not a good indicator of radiation damage due to the confounding effect of senescent cells. Currently, the growth kinetics of tumour spheroids is the most studied end-point in drug and therapy screening studies. After irradiation, a large portion of cells in the spheroids will enter the senescent stage, but still contribute to the growth in spheroid size, making this method less relevant for radiobiological studies. As senescent cells are generally less dense and as FLASH irradiation was shown to produce less senescent cells (Ref. 20), it is possible that the difference between FLASH and CONV irradiation can be evaluated by measuring the weight or density of the tumour spheroids (Ref. 135) or analysing the senescent biomarkers using IHC (Ref. 136). In addition, high content imaging with important biomarkers for different stages of cell cycles and DDR signalling may also highlight the biological difference between FLASH and CONV irradiation (Refs 137, 138). Overall, spheroids provide a simple 3D tumour model to study the FLASH effect under a hypoxic tumour microenvironment.

### Organoids

Organoids are 3D miniature and self-organised cultures showing physiological micro-anatomy. They can be derived from adult stem cells, embryonic or pluripotent stem cells. Unlike spheroids, organoids consist of heterogeneous cell types including stem cells or progenitor cells that are crucial for normal tissue response to radiation damage. The organoid culture also requires extracellular matrix and growth factors. The advantage of organoid is that they can be passaged *in vitro* and cryopreserved, facilitating biobanking for further preclinical studies. The application of organoids in FLASH-RT has not yet been reported, but they have been used to study the radiosensitivity and radiation damage of normal tissues. Martin *et al*. showed that small and large intestine organoids were able to recapitulate the radiosensitivity profile of the intact organ (Ref. 139). Das *et al*. used human induced pluripotent stem cell-derived cerebral organoids to study the radiation-induced DNA repair (Ref. 140). Martinez *et al*. used parotid salivary gland organoids to study the radiation response (Ref. 141). Mammary organoids have also been used to study the immune cell recruitment (Ref. 142). Patient-derived tumour specimens can also be a source for tumour organoids (tumouroids). Tumouroids can reflect the genetic and phenotypic heterogeneity of their original sources, making them a valuable tool for personalised medicine (Refs 143–145). In addition to cellular phenotype, organoids can mimic the hypoxic microenvironment of the original tissues. Hubert *et al*. showed that patient-derived glioblastoma organoids demonstrated hypoxia gradient and cancer stem cell heterogeneity similar to *in vivo* conditions (Ref. 146). Multi-parametric imaging of oxygen tension and cell cycle stage has also been developed in intestinal organoids (Ref. 147). Therefore, organoids can be a useful tool to study the dynamic cellular response in tumours and normal tissues after radiation. Both tumouroids and normal tissue organoids can be derived from the same patients, which could allow for the development of safe and efficient personalised treatments using this protocol.

### Tissue slice cultures

Tissue slice cultures, or organotypic culture, are generated by cutting non-fixed normal tissues or tumours into thin slices. These slices can preserve the morphology and microenvironment of the original tissues for several days. They have been commonly used in drug screening and in a few radiobiological studies. Suckert *et al*. used tumour slices of head and neck squamous cell carcinoma and slice culture of adult mice brain to study the radiation response of proton beam RT (Ref. 148). They found that tumour slice culture demonstrated DNA damage and morphology results similar to the *in vivo* condition, while brain slice culture failed to reveal relevant radiation response, potentially due to change in cell morphology and phenotype after long term culture. On the other hand, the use of neonatal brain slice culture has proven more successful in maintaining the morphology and phenotype after long term culture (Ref. 149). With the preservation of immune cells in the tumour microenvironment, tumour slice cultures have been commonly used to study immunotherapy. Innate immunity can be induced by PD-1 inhibitors on patient-derived lung tumour slices (Ref. 150). In addition, the use of patient-derived lung tumour slices can be predictive of the immunotherapy outcome (Ref. 151). In summary, tissue slice cultures can be used to study short term radiation effect on the bona fide cellular microenvironment and in physiological conditions.

### Organ-on-Chip models

One of the major limitations in 2D and 3D cell culture models is the ability to simulate flow and relevant mechanical properties (e.g. shear stress and tensile strain). Organ-on-chip models provide the solution to incorporate fluid dynamics and biomechanics into the culture systems. Organ-on-chip models are created by culturing cells of 2D or 3D formats in a microfluidic device that can control the physical or chemical microenvironment of the cells. Though it has not been widely applied in studying FLASH-RT, several projects have been established to use organ-on-chip systems as a countermeasure of radiotoxicity (Ref. 152). Patient-derived head and neck squamous cell carcinoma has been used in an organ-on-chip model to assess the radiation-induced cell death and could predict the clinical outcome (Refs 153, 154). Radiation-induced cell death and protection by radioprotectors were also demonstrated on a Gut-on-a-Chip model (Ref. 155). An oxygen gradient can be easily created in the organ-on-chip model by using chemical oxygen quenchers in the medium or pumping nitrogen/carbon dioxide through the device (Refs 156, 157). It is also possible to link multiple organ-on-chip models together to create a body-on-chip system. This may also open the door to studies of systemic radiation effects such as long-range bystander (Ref. 158) and abscopal effects, which is not possible using simpler *in vitro* models. The highly tailorable nature of organ-on-chip models make them a versatile tool to study the effect of both cellular and environmental factors following irradiation. Biochemical sensors can also be integrated into the organ-on-chip systems to provide *in situ* monitoring of cellular and microenvironmental parameters.

### Engineered tissue models by 3D bioprinting

3D bioprinting allows for the construction of complex multi-cell models layer by layer, with high flexibility and precision. The cells are printed into bio-inks, which are generally biocompatible materials. The bio-inks determine the physical and chemical microenvironment of the printed tissues. The choice of bio-ink is important when using the engineered tissue for modelling radiation damage response as it can affect the cellular response (Ref. 159). Vascularised engineered tissues can be created by 3D bioprinting to simulate oxygen and nutrient gradients (Ref. 160). Oxygen released bio-ink can also be used to modulate the local oxygen tension inside the engineered construct (Ref. 161). Today 3D bioprinting focuses mostly on regenerative medicine but with the development of high throughput printing technology, it is expected that the engineered tissues can also be used for tumour/normal tissue modelling to study the FLASH effect. The combination of patient-derived cells and stimuli-responsive bio-inks, which can change the physical or biochemical environment, offer a platform to study the dynamic response of physiologically relevant tissues following FLASH irradiation.

## Concluding remarks/translational implications

The FLASH effect is an intriguing phenomenon that is currently being studied by many research labs around the world. The magnitude of the effect is such that it could be a ‘game changer’ for the treatment of many tumours. However, as we do not yet understand the effect or know the mechanisms behind the effect, we cannot translate it into the clinic in an optimal way. Consequently, more preclinical studies are needed. Specifically, *in vitro* studies to complement the more challenging and expensive *in vivo* studies. Here, we have described several *in vitro* assays that have been used or could be used to finally elucidate the mechanisms behind the FLASH effect. The doses often required to show a significant FLASH effect (⩾10 Gy) make some of the mentioned assays more suitable than others, e.g. the comet assay compared to *γ*H2AX.

FLASH-RT is a very promising new radiotherapy technique that we would like our cancer patients to benefit from as soon as possible. However, a successful clinical translation of the technique hinges on a better understanding of the FLASH sparing effect of normal tissues. If we understand this phenomenon, we can optimise our FLASH treatments to maximise the effect, e.g. by using hypofractionated approaches (Ref. 17), non-homogeneous irradiation (Ref. 162), and combination with drugs that enhances the effect (Ref. 163). Preclinical assays, such as the ones described above, will be essential tools in identifying the radiobiological mechanisms behind the effect and finally being able to fully exploit the benefits associated with FLASH-RT.
